# The Book World of Medicine and Science

**Published:** 1902-12-06

**Authors:** 


### The Book World of Medicine and Science.

A Concise History of Small-pox and Vaccination in Europe. By E. J. Edwardes, M.D., etc. Cr. 8vo., cloth. (London: H. K. Lewis. Price 2s. 6d. net.)

This is a readable little book, in spite of the fact that it is necessarily full of statistics. Beginning with a short account of small-pox as it existed before the introduction of vaccination, it describes Jenner's work, and then gives the history of small-pox and vaccination from that date up to the present time. Among the points which stand out most clearly are, first, that before the introduction of vaccination small-pox was essentially a disease of childhood, and, secondly, that after vaccination had become pretty general, small-pox was almost exclusively met with in adults, in whom the immunity conferred by early vaccination had passed off. Dr. Edwardes uses the above facts as the basis of a strongly-worded plea for the compulsory revaccination of all children at the age at which they go to school; and he criticises severely the present state of the law in England; for, as he puts it, we are as certain that revaccination is necessary as we are that primary vaccination is necessary. He suggests that the word "obligatory" should be used instead of "compulsory," but it is not likely that the change would conciliate anyone, and it would certainly have no effect upon the out-and-out opponents of vaccination.

Suggested Standards of Purity for Foods and Drugs. By C. G. Moor, M.A., F.I.C., F.C.S., pp. 260. Crown 8vo. (London: Baillière, Tindall and Cox. 1902. Price 7s. 6d. net.)

This is a well-intentioned work. It seeks to suggest certain limits or standards as indicating the genuineness of various foods and drugs. A spirit is abroad which seeks improvement in our methods of testing, reform in our analytical procedure, and greater precision in estimating the purity of medicines and articles of diet. Mr. Moor recognises that the data which he presents are necessarily tentative, but the records of the results of actual investigation of many preparations goes far to bring near a day when more perfect evidence shall be available. The work should prove of much service to public analysts and pharmacists, and all interested in the maintenance of a high standard of excellence in pharmaceutical and dietetic preparations will find much that is highly suggestive in these pages. The scope of the work is necessarily limited, and unfortunately what appear to be almost sins of omission are peculiarly noticeable. Thus while we have sections on butter there is no reference to beer, and the extremely important matter of preservatives receives no due consideration. The author expresses a hope that in a further edition the work may be rendered more complete, and he modestly solicits correction "either publicly or privately."

Anæsthetics: a Practical Handbook. By J. Blumfeld, M.D.Cantab., Assistant Anæsthetist to St. George's Hospital; Anæsthetist to the Grosvenor Hospital for Women and Children. pp. 109, with 9 illustrations. (London: Baillière, Tindall and Cox. 1902. 2s. 6d. net.)

This eminently practical little manual forms the latest addition to the useful "Medical Monograph Series," edited by Dr. David Walsh. It aims at presenting a concise and lucid account of the principles upon which the common anæsthetics are selected and administered, and endeavours to furnish serviceable descriptions of the practical details involved. The work is well designed and satisfactorily executed. A good anæsthetist cannot, of course, be made by mere book instruction; he must gain his knowledge by clinical observation, wide experience, and thorough training in the management of all varieties of case. Still as a common-sense introduction to the rational study of anæsthetics for the student, and a practical guide to the general practitioner, this brochure should prove of much service. The nature and action of the common anæsthetics, nitrous oxide, ether and chloroform receive careful consideration, and while debatable points are wisely avoided, much sound advice is given. Valuable guidance is afforded on the use of anæsthetics in succession and in mixtures, the choice of an anæsthetic in various conditions, on the dangers and troubles incidental to anæsthesia, on the question of position, on the administration of anæsthetics to children, the preparation of the patient, and the character of after effects. The various sections are well arranged, the material presented is conveniently grouped, the type is clear, and there is a good index. We wish a larger number of illustrations had been given, and a bibliography of the more important modern works would have been of service for reference. A more careful proof reading would have avoided the blunder of several typographical errors.

Aids to Dental Anatomy and Physiology. By Arthur S. Underwood, M.R.C.S., L.D.S. Second edition. (London: Baillière, Tindall and Cox. 1902. Price 2s. 6d. cloth; 2s. paper.)

Although this is a small book, it gives a useful and readable account of the subject dealt with, and one quite sufficiently full to meet the requirements of the ordinary student. The first chapter which is on the subject of the nature of the teeth contains much useful and suggestive information, serving well to show how each animal's teeth are exactly fitted to their environment, and how perfectly adapted they are to the work they have to do. As the author says, so thoroughly has evolution provided appropriate apparatus for the various families of the animal kingdom that it is comparatively easy from a glance at an animal's teeth to decide what were its habits, its general characteristics, and its surrounding circumstances. The succeeding chapters deal in succession with development, calcification, the histology of the various tissues, the development of the mouth in relation to the teeth, and the teeth of man in particular. Finally, there is a chapter on practical microscopy, which will be found very useful, the cutting of sections in such hard tissues being somewhat special work. Thus the whole subject is gone through. It will be at once understood that to compress so large a subject into so small a compass, it has been necessary to keep pretty closely to the matter in hand. Still room is found for suggestions which render it interesting reading, and the student who goes carefully through its pages ought not only to obtain a good general survey of the histology and physiology of the teeth, but to gain many ideas in regard to their relation to the organism at large which will be useful to him in various ways.

